# Analytical performances of the AMPLIQUICK^®^ Respiratory Triplex assay for simultaneous detection and differentiation of SARS-CoV-2, influenza A/B and respiratory syncytial viruses in respiratory specimens

**DOI:** 10.1371/journal.pone.0262258

**Published:** 2022-01-05

**Authors:** Ralph-Sydney Mboumba Bouassa, Serge Tonen-Wolyec, David Veyer, Hélène Péré, Laurent Bélec

**Affiliations:** 1 Ecole Doctorale d’Infectiologie Tropicale, Franceville, Gabon; 2 Laboratoire de Virologie, Hôpital Européen Georges Pompidou, Assistance Publique-Hôpitaux de Paris, Paris, France; 3 Faculty of Medicine and Pharmacy, University of Kisangani, Kisangani, The Democratic Republic of the Congo; 4 Université de Paris, Sorbonne Paris Cité, Paris, France; Loyola University Health System, UNITED STATES

## Abstract

Although patients infected with severe acute respiratory syndrome coronavirus 2 (SARS-CoV-2), influenza A, influenza B and respiratory syncytial virus (RSV) show comparable or very similar manifestations, the therapeutic approaches of these respiratory viral infections are different, which requires an accurate diagnosis. Recently, the novel multiplex real-time reverse transcription-polymerase chain reaction assay AMPLIQUICK^®^ Respiratory Triplex (BioSynex SA, Illkirch-Graffenstaden, France) allows simultaneous detection and differentiation of SARS-CoV-2, influenza A, influenza B, and RSV in respiratory tract samples. We herein evaluated the performance of the AMPLIQUICK^®^ Respiratory Triplex for the detection of the four viruses in respiratory specimens, using Allplex™ Respiratory Panel 1 and 2019-nCoV assays (Seegene, Seoul, Korea) as reference comparator assays. A total of 359 archived predetermined respiratory samples, including 83, 145, 19 and 95 positive specimens for SARS-CoV-2, influenza A, influenza B and RSV respectively, were included. The AMPLIQUICK^®^ Respiratory Triplex showed high concordance with the reference assays, with an overall agreement for SARS-CoV-2, influenza A, influenza B, and RSV at 97.6%, 98.8%, 98.3% and 100.0%, respectively, and high κ values ranging from 0.93 to 1.00, indicating an almost perfect agreement between assays. Furthermore, high correlations of cycle threshold (C_t_) values were observed for positive samples of the four viruses between the AMPLIQUICK^®^ Respiratory Triplex and comparator assays, with an overall high agreement between C_t_ values assessed by Bland-Altman analyses. In conclusion, these observations demonstrate that the multiplex AMPLIQUICK^®^ Respiratory Triplex is a reliable assay for the qualitative detection and differentiation of SARS-CoV-2, influenza A, influenza B, and RSV in respiratory specimens, which may prove useful for streamlining diagnostics during the winter influenza-seasons.

## Introduction

Since the emergence of the coronavirus disease 2019 (COVID-19) caused by the Severe Acute Respiratory Syndrome coronavirus 2 (SARS-CoV-2), many countries face now a threat of concurrent circulation of SARS-CoV-2 with more common other respiratory viruses such as influenza A and B, and the respiratory syncytial virus (RSV) [1–3]. These viruses share several similarities in term of clinical symptoms, transmission routes and seasonal distribution [4, 5], making challenging the differential diagnosis of these viral infections by healthcare providers. In addition, co-infection with SARS-CoV-2 and influenza as well as other concurrent respiratory viruses have already been reported [6–17], with influenza A and RSV being the commonest co-viruses in some settings [2].

Given that the treatment and management approaches for these infections are quite different, this raises the problem of defining a diagnostic algorithm that is sufficiently robust and reliable to be able to discriminate between these different viral infections. Clinical microbiology and public health laboratories are likely facing pressure to offer parallel testing for these viruses from a biological specimen of the same patient, optimally using assays allowing to detect a single target, either SARS-CoV-2, influenza A and B, or RSV. However, positivity for one target does not rule-out infection with another respiratory virus [6–8, 10, 11, 15, 17]. Conversely, clinicians cannot rule out a SARS-CoV-2 infection by ruling in other respiratory pathogens during the COVID-19 pandemic [12]. Therefore, it is crucial to detect the clinical etiology to accurately rule out SARS-CoV-2 or other upper respiratory viral infections and to appropriately monitor coinfections in patients with COVID-19.

An optimal diagnostic algorithm for testing patients with influenza-like disease is a multiplex assay that combines the four targets to test for SARS-CoV-2, influenza A, influenza B, and RSV in the same specimen, in a single shut. To assist global efforts in the fight against the spread of COVID-19 and influenza during the upcoming influenza-season, a number of manufacturers are modifying existing assays to allow for multiplex testing of SARS-CoV-2 and influenza A, influenza B, and RSV simultaneously [18–24].

In this study, we evaluated the analytical performances of the novel multiplex real-time reverse transcription-polymerase chain reaction (rtRT-PCR) assay AMPLIQUICK^®^ Respiratory Triplex developed by BioSynex SA (Illkirch-Graffenstaden, France) for the simultaneous detection and differentiation of SARS-CoV-2, influenza A, influenza B, and RSV in respiratory specimens.

## Materials and methods

### Clinical specimen collection and reference molecular testing for respiratory viruses

This monocenter, retrospective study included a total of 442 nasopharyngeal specimens or broncho-alveolar lavage samples from adult patients (≥ 18 years old) suffering from influenza-like signs and symptoms from 1st October 2008 to 30th August 2020. Globally, the study population of this study was constituted by a total of 83 specimens from patients with confirmed COVID-19 positive tests obtained during the first and second waves of the SARS-CoV-2 epidemic in France from March to August 2020. In addition, a total of 164 cryopreserved specimens from patients positive for influenza A (145 specimens) or B infections (19 specimens) and archived during the five 2015–2020 winter seasons were also included. Likewise, 95 cryopreserved specimens from patients with respiratory disease associated with RSV infections tested mainly during the RSV outbreaks in Paris in 2008–2009 and 2012–2013 winter, and the other during the two 2018–2020 autumn-winter seasons were included in this study. All patients infected by RSV were inpatients from the geriatric ward of our institution, or patients admitted to the emergency unit for acute respiratory distress; all were above 65-year-old. All samples positive for influenza or RSV during winter 2019–2020 were negative for SARS-CoV-2 RNA. Finally, 100 archived respiratory specimens collected before the SARS-CoV-2 epidemic in France and negative for influenza A, influenza B and RSV were used as negative controls.

Respiratory secretions corresponded mainly of nasopharyngeal specimens including nasopharyngeal aspirates and nasopharyngeal flocked swabs collected by a nurse or physician using standardized methods, or broncho-alveolar lavage. After sampling, the nasopharyngeal swab was discharged into a vial containing 3 mL of virus transport medium (Xpert^®^ Viral Transport medium, Cepheid, Sunnyvale, CA, USA). The respiratory specimens were then aliquoted, for reference molecular testing of respiratory viruses and for further conservation at −80°C until use.

Molecular testing of respiratory viruses was carried out after automatic nucleic acid extraction with the STARMag 96x4 Universal Cartridge Kit (Seegene Seoul, Korea) using the Allplex™ Respiratory Panel 1 assay (Seegene), a multiplex one-step rtRT-PCR that detects common respiratory viruses, chosen as the reference rtRT-PCR for influenza A/B and RSV RNA detection, as previously described [25]. Molecular testing of SARS-CoV-2 RNA used the multiplex real-time PCR Allplex™ 2019-nCoV assay (Seegene), a reliable molecular assay which can simultaneously detect 3 coronavirus target genes, including envelope protein gene (E), RNA-dependent RNA polymerase gene (RdRP) and nucleocapsid protein gene (N), as described [26]. Real-time RT-PCR was carried out with CFX96™ Real-Time PCR Detection System (Bio-Rad Laboratories, Hercules, CA, USA), according to the manufacturer’s instructions. Extraction and PCR setup were controlled with Seegene Launcher IVD (Seegene) and results were automatically analyzed using Seegene Viewer IVD software (Seegene). Individual cycle threshold (C_t_) values were recorded for each target gene.

### Simultaneous detection of SARS-CoV-2, influenza A, influenza B, and RSV by AMPLIQUICK^®^ Respiratory Triplex

Nucleic acid extraction was performed from 300 μL thawed aliquots of the eluted nasopharyngeal swab samples, nasopharyngeal aspirates and broncho-alveolar lavages using automated nucleic acid extraction EX3600 extractor (Liferiver & Shanghai ZJ Bio-Tech Co., Shanghai, China) with the Liferiver^®^ Viral RNA Extraction kit (Liferiver & Shanghai ZJ Bio-Tech Co.; reference NE-0044).

The AMPLIQUICK^®^ Respiratory Triplex (BioSynex SA) is a multiplex rtRT-PCR assay for the simultaneous detection and differentiation of SARS-CoV-2, influenza A, influenza B, and RSV. The kit is composed of 96-well microplate pre-filled with the master mix containing dNTPs, MgCl_2_, fluorescent primers and probes, Taq polymerase and reverse transcriptase enzymes, and reaction buffer. The assay can simultaneously detect 2 coronavirus target genes, including the SARS-like (including SARS-CoV-2, SARS-CoV, bat SARS-like coronavirus) conserved region of envelope protein gene (E), and RNA-dependent RNA polymerase gene (ORF1ab of RdRP gene), the gene encoding the matrix protein (M) of influenza A, the gene encoding the hemagglutinin (HA) of influenza B, and the gene encoding the matrix protein (M) of RSV A and RSV B, providing individual C_t_ values for each target gene. The detection of amplified virus cDNA fragment is performed in fluorimeter channels FAM for E gene of SARS-CoV-2 and M gene of influenza A, HEX for RdRP gene and HA gene of influenza B, and Cy5 for the M gene of RSV. In addition, the kit contains a system to identify possible PCR inhibition by measuring the Cy5 fluorescence of the human RNase P gene as internal control. This assay was performed on the CFX96™ Real-Time PCR Detection System (Bio-Rad Laboratories) according to the manufacturer’s instructions. The experiment and result interpretation were carried out according to the manufacturer’s protocol.

### Clinical performance comparison using clinical samples

The Allplex™ Respiratory Panel 1 and Allplex™ 2019-nCoV assays were used as reference comparator assays. Seegene’s Allplex Respiratory Panel 1 assay was shown to be highly sensitive, specific, and suitable for detection of influenza A and B and RSV [25].

Specimens with discordant results between the AMPLIQUICK^®^ Respiratory Triplex and comparator assays were further resolved with third alternative assays, including the multiplex rtRT-PCR Novel Coronavirus (2019-nCoV) Real-Time Multiplex RT-PCR Kit (Detection for 3 Genes) (Liferiver & Shanghai ZJ Bio-Tech Co.) for SARS-CoV-2 and the Xpert^®^ Xpress Flu/RSV kit (Cepheid, Sunnyvale, CA, USA) for influenza A, influenza B and RSV.

### Statistical analysis

Data were entered into an Excel database. Means and standard deviations (SD) were calculated for quantitative variables and proportions for categorical variables. The results were presented along with their two-sided 95% confidence interval (CI) using the Wilson score bounds for categorical variables [27].

Firstly, the results of SARS-CoV-2, influenza A, influenza B and RSV detection by the multiplex molecular detection of SARS-CoV-2 and other respiratory viruses (Allplex™ assays) were used as the reference standard to estimate the sensitivity and specificity of the AMPLIQUICK^®^ Respiratory Triplex, with corresponding 95% CI. The concordance between the AMPLIQUICK^®^ Respiratory Triplex and the reference comparator assays was assessed by percent agreement corresponding to the observed proportion of identical results between AMPLIQUICK^®^ Respiratory Triplex and the comparator assays. The reliability between the AMPLIQUICK^®^ Respiratory Triplex and the comparator assays was estimated by Cohen’s κ coefficient [28], and the degree of agreement was determined as ranked by Landlis and Koch [29]. The accuracy of the AMPLIQUICK^®^ Respiratory Triplex to correctly diagnose SARS-CoV-2, influenza A, influenza B and RSV was estimated by Youden’s J index (J = sensitivity + specificity − 1) [30]. Positive predive value (PPV) and negative predictive value (NPV) were calculated for the AMPLIQUICK^®^ Respiratory Triplex according to the Bayes’s formula, using the overall prevalence of 16.2% on 17^th^ November 2020 of SARS-CoV-2-RNA positivity in COVID-19-suspected patients during the peak of the second wave of the COVID-19 epidemic in France [Santé publique France 2020; https://www.santepubliquefrance.fr/], and the observed prevalences of influenza (38.0%) and RSV (3.7%) infections in adults admitted to hospital in France with influenza-like illness during three influenza-seasons (2012–2015) in France [31].

Secondly, further correlations and quantitative agreement analyses were assessed. First, correlations between the C_t_ values of target genes obtained by the AMPLIQUICK^®^ Respiratory Triplex and the reference Allplex™ and rtRT-PCRs were established by the Passing-Bablok nonparametric linear regression method [32]. Secondly, the agreement between the AMPLIQUICK^®^ Respiratory Triplex and Allplex™ rtRT-PCRs was depicted by difference plots as proposed by Bland and Altman [33, 34]. The Bland-Altman analyses were carried out to calculate the mean of absolute bias and limits of agreement, respectively, corresponding to the 95% CI [± 1.96 × standard deviation (SD)] of the mean absolute bias of all paired measurements [34]. The *P*-value < 0.05 was considered as statistically significant.

Statistical analyses were performed using Method Validator software version 1.1.9.0. (Philippe Marquis, France) and XLSTAT v19.1 software (Addinsoft, Paris, France).

### Ethical statement

The study was conducted as a continuous quality improvement program for SARS-CoV-2, influenza and RSV diagnosis and care, consistent with institutional policies on the accreditation of medical biology laboratories [35]. The dataset was completely anonymous and did not contain any identifiable personal health information. Our non-interventional study was carried out in accordance with the Declaration of Helsinki without extra sample collection compared to usual procedures. In particular, respiratory specimens were obtained only for standard diagnostic following medical prescriptions and care. Under these conditions, the study was exempted from informed consent application, according to the French public health code (Code de la Santé Publique, article L 1121–1.1; https://www.legifrance.gouv.fr/). Data analyses were carried out using the anonymized database.

## Results

The Table 1 depicts the analytical performances of the AMPLIQUICK^®^ Respiratory Triplex to simultaneously detect SARS-CoV-2, influenza A, influenza B and RSV from of a total of 442 respiratory specimens including 83 samples positive for SARS-CoV-2 RNA by the reference test (Allplex™ Respiratory Panel 1 and Allplex™ 2019-nCoV assays, Seegene), 259 archived respiratory samples positive for influenza A, influenza B or RSV, and 100 samples collected before the SARS-CoV-2 epidemic in France and negative for influenza A and B and RSV.

**Table 1:** Analytical performances of the AMPLIQUICK® Respiratory Triplex (BioSynex SA) for the simultaneous detection of SARS-CoV-2, influenza A, influenza B and RSV by comparison to the results from the Allplex™ Respiratory Panel 1 and Allplex™ 2019-nCoV assays (Seegene) used as reference tests.

				AMPLIQUICK^®^ Respiratory Triplex									
				N	Positive	Negative	Sensitivity *(% [95% CI*])^μ^	Specificity *(% [95% CI])*	Agreement^a^ *(% [95% CI])*	Concordance^b^ *(% [95% CI])*	Youden’ J index^c^ (% [95% CI])	PPV^d^ *(% [95% CI])*	NPV^d^ *(% [95% CI])*
**Reference assays**	**SARS-CoV-2**	Positive	All C_t_^$^ values	83	81	2	97.6 [95.7–98.7]	100 [99.1–99.9]	99.5 [98.3–99.9]	98.4 [96.7–99.2]	97.6 [95.7–98.7]	100 [99.1–100]	99.5 [98.3–99.9]
			**≤ 33**	47	47	0	100 [99.1–100]	100 [99.1–100]	100 [99.1–100]	100 [99.1–100]	100 [99.1–100]	100 [99.1–100]	100 [99.1–100]
			**> 33**	36	34	2	94.4 [91.7–96.3]	100 [99.0–100]	99.5 [98.1–99.9]	97.0 [94.8–98.3]	94.4 [91.7–96.3]	100 [99.0–100]	98.9 [97.3–99.5]
		Negative		359	0	359	-	-	-	-	-	-	-
	**Influenza A**	Positive		145	142	3	97.9 [95.2–99.1]	100 [98.4–100]	98.8 [96.5–99.6]	97.4 [94.5–98.8]	97.9 [95.2–99.1]	100 [98.4–100]	98.7 [96.3–99.5]
		Negative		100	0	100							
	**Influenza B**	Positive		19	17	2	89.5 [82.7–93.8]	100 [96.9–100]	98.3 [94.0–99.5]	93.3 [87.3–96.6]	89.5 [82.7–93.8]	100 [96.9–100]	93.5 [87.6–96.7]
		Negative		100	0	100							
	**Influenza A/B**	Positive		164	159	5	96.9 [94.0–98.4]	100 [98.6–100]	98.1 [95.6–99.2]	96.0 [92.9–97.8]	96.9 [94.0–98.4]	100 [98.6–100]	98.1 [95.6–99.2]
		Negative		100	0	100							
	**RSV**	Positive		95	95	0	100 [98.1–100]	100 [98.1–100]	100 [98.1–100]	100 [98.1–100]	100 [98.1–100]	100 [98.1–100]	100 [98.1–100]
		Negative		100	0	100							

^a^ Agreement = TP + TN / TP+FP+TN+FN, expressed in percentage

^b^ The Cohen’s κ coefficient calculation was used to estimate the concordance [28] and interpreted according the Landis and Koch scale [29], as follows: < 0 as indicating no agreement, 0–0.20 as slight, 0.21–0.40 as fair, 0.41–0.60 as moderate, 0.61–0.80 as substantial, and 0.81–1 as almost perfect concordance

^c^ The accuracy of the AMPLIQUICK^®^ Respiratory Triplex platform to correctly diagnose SARS-CoV-2 infection was estimated by Youden’s J index (J = sensitivity + specificity − 1) [30]

^d^ PPV and NPV were calculated according to the Bayes’s formulae, by taking into account the official reported prevalence of SARS-CoV-2-RNA positivity in COVID-19-suspected patients in Paris’s area, France, of 16.2% on 17^th^ November 2020 [Santé publique France 2020; https://www.santepubliquefrance.fr/], as well the observed prevalences of influenza (38.0%) and RSV (3.7%) infections in adults admitted to hospital in France with influenza-like illness during the 2012–2015 influenza-seasons in France [31]

^μ^ 95% confidence intervals in brackets were calculated by using the Wilson score bounds

^$^ The C_t_ values of RdRP gene detection by the rtRT-PCR Allplex™ 2019-nCoV assay (Seegene) were used to classify nasopharyngeal samples according to their level of SARS-CoV-2 RNA excretion; Ct < 33 was taken as threshold of high SARS-CoV-2 RNA excretion, as previously stated [16, 36, 37].

CI: Confidence interval; C_t_: Cycle threshold; NPV: Negative predictive value; PPV: Positive predictive value; rRT-PCR: real-time reverse transcription-polymerase chain reaction; RSV: Respiratory syncytial virus.

### Analytical results

The 83 respiratory specimens positive for SARS-CoV-2 RNA showed detectable E and RdRP genes by Allplex™ 2019-nCoV assay, whereas only 81 and 68, respectively, were positive for RdRP and E genes by AMPLIQUICK^®^ Respiratory Triplex. All samples positive for E gene were also positive for RdRP gene. Finally, according to the manufacturer’s instructions of the AMPLIQUICK^®^ Respiratory Triplex (BioSynex SA), human RNase P-, E- and RdRP- positive samples as well as human RNase P- and RdRP- positive samples were considered as positive for SARS-CoV-2 RNA. Two SARS-CoV-2-positive samples found negative by AMPLIQUICK^®^ Respiratory Triplex, showing C_t_ of 37.8 and 39.1, respectively, in E gene, and C_t_ of 39.7 and 40.5, respectively, in RdRP gene by Allplex™ 2019-nCoV assay, were further tested positive by the 2019-nCoV Multiplex rtRT-PCR assay for SARS-CoV-2 RNA detection (Liferiver & Shanghai ZJ Bio-Tech Co.). All the 259 archived predetermined respiratory samples positive for influenza A, influenza B or RSV, and the 100 samples collected before the SARS-CoV-2 epidemic in France and negative for influenza A and B and RSV, were negative for SARS-CoV-2 RNA by AMPLIQUICK^®^ Respiratory Triplex.

Among 145 respiratory specimens positive for influenza A and 19 positive for influenza B by reference Allplex™ Respiratory Panel 1 assay, 142 and 17, respectively, were found concordantly positive by AMPLIQUICK^®^ Respiratory Triplex. The 3 influenza A-positive and 2 influenza B-positive samples found negative by AMPLIQUICK^®^ Respiratory Triplex showed C_t_ of 35.5, 37.0, 37.9, 35.0 and 33.7, respectively, by Allplex™ Respiratory Panel 1 assay, and were further tested positive by Xpert^®^ Xpress Flu/RSV kit. All 95 respiratory specimens positive for RSV by reference Allplex™ Respiratory Panel 1 assay, were found positive by AMPLIQUICK^®^ Respiratory Triplex. There was 5 cases of influenza A and RSV co-infections, similarly detected by the reference Allplex™ Respiratory Panel 1 assay and by the AMPLIQUICK® Respiratory Triplex. Finally, all 100 samples collected before the SARS-CoV-2 epidemic in France and negative for influenza A/B and RSV by reference Allplex™ assay were found negative by AMPLIQUICK^®^ Respiratory Triplex.

### Agreement between AMPLIQUICK^®^ Respiratory Triplex and comparator assays

For the detection of SARS-CoV-2 RNA, the AMPLIQUICK^®^ Respiratory Triplex showed high sensitivity, specificity, PPV and NPV of 97.6%, 100.0%, 100.0% and 99.5%, respectively, high or almost perfect agreement (99.5%), reliability assessed by Cohen’s κ coefficient (0.98), and accuracy assessed by Youden’s J index (87.6%) to detect SARS-CoV-2 RNA.

The analytical performances of the AMPLIQUICK® Respiratory Triplex were also stratified according to the C_t_ values of the N gene detected by reference Allplex™ 2019-nCoV assay, considering C_t_-related criteria of high (C_t_≤33) SARS-CoV-2 RNA excretion [16, 36, 37]. In case of high viral excretion, the analytical performances of the AMPLIQUICK® Respiratory Triplex remained excellent. In the event of moderate or very low viral excretion (Ct>33), the sensitivity, concordance and accuracy of the AMPLIQUICK^®^ Respiratory Triplex dropped slightly, while the specificity, PPV and NPV remained constant.

For influenza A and B infection as well as RSV infection, the AMPLIQUICK^®^ Respiratory Triplex showed similarly high sensitivity, specificity, PPV, NPV, agreement, reliability and accuracy, with an overall agreement for influenza A, influenza B, and RSV at 98.8%, 98.3% and 100.0%, respectively, and high κ values ranging from 0.93 to 1.00, indicating an almost perfect agreement between assays.

### Association of C_t_ values between AMPLIQUICK^®^ Respiratory Triplex and comparator assays

**Fig 1:**
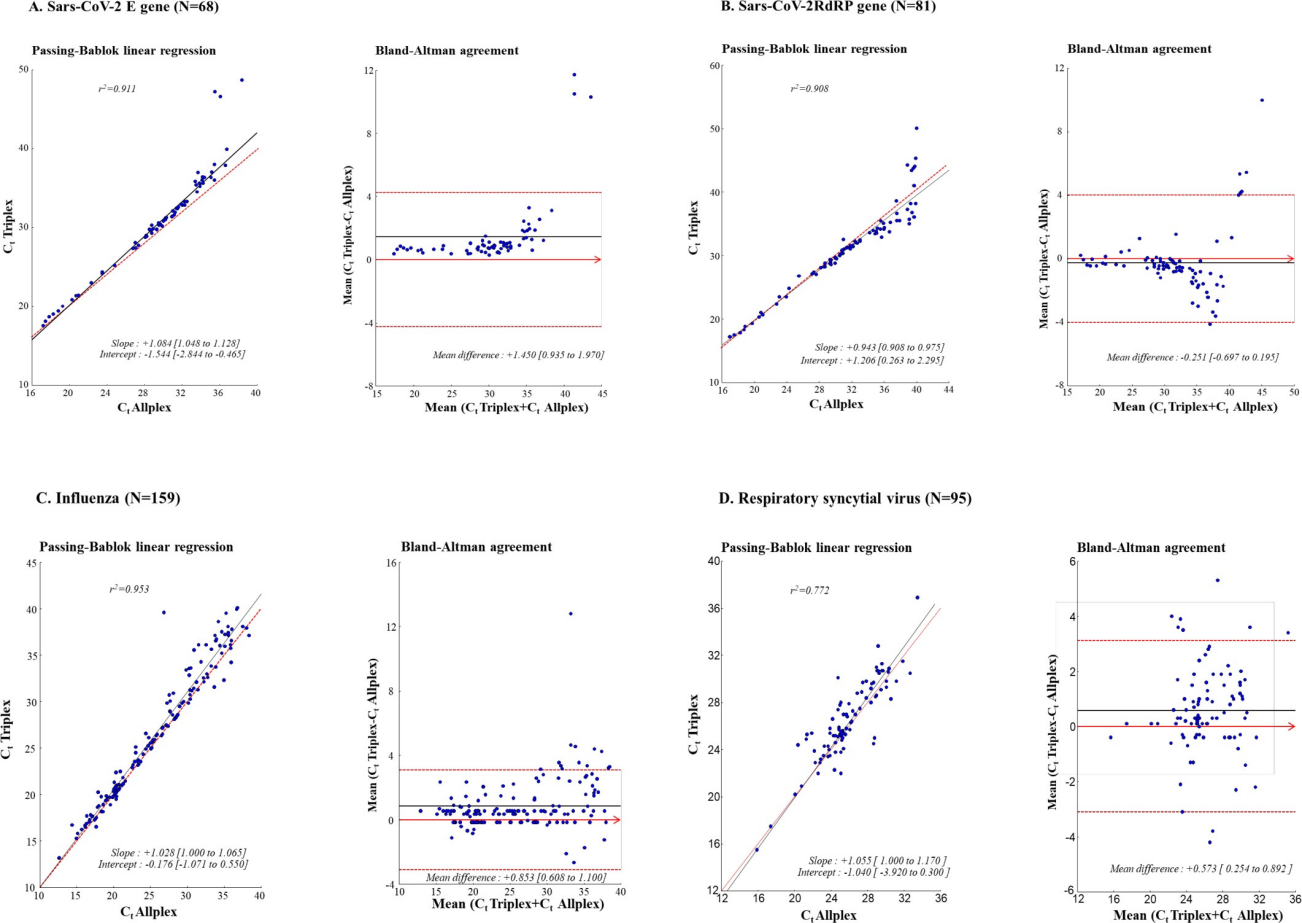
Passing-Bablok and Bland-Altman analyses of C_t_ values obtained by AMPLIQUICK^®^ Respiratory Triplex rtRT-PCR (BioSynex SA) and comparator assays. Passing-Bablok nonparametric linear regression curves between the C_t_ values obtained by the AMPLIQUICK^®^ Respiratory Triplex rtRT-PCR (BioSynex SA) and the Allplex™ Respiratory Panel 1 and Allplex™ 2019-nCoV assays (Seegene), used as reference comparator assays, and Bland-Altman analyses on the relative differences between the C_t_ values obtained by the AMPLIQUICK^®^ Respiratory Triplex and the reference Allplex™ assays, calculated from C_t_ values results of paired detection of SARS-CoV-2 E gene (**A.**), SARS-CoV-2 RdRP gene (**B.**), influenza A and B RNA (**C.**) and RSV RNA (**D.**) in archived predetermined clinical respiratory samples. For Passing-Bablok nonparametric linear regression curves, the diagonal dotted line depicts the ideal line (*i*.*e*. no bias), whereas the full line indicates the regression line of the distribution. For the Bland-Altman analyses, the full line depicts the mean relative difference, the dotted lines indicate the superior and inferior limits of agreement, and the arrow is the x abscise axis. Quantitative results are C_t_ values for each virus or target genes detected. Only results from samples positive by both AMPLIQUICK^®^ Respiratory Triplex and reference Allplex™ assays were used for analyses. C_t_: Cycle threshold.

The non-parametric Passing-Bablok regression analyses between the C_t_ values of samples positive for SARS-CoV-2, influenza A, influenza B or RSV obtained by both the AMPLIQUICK^®^ Respiratory Triplex and the Allplex™ comparator assays are shown in Fig 1. There was a remarkably high correlation between C_t_ values of positive samples by the two assays for SARS-CoV-2 (r^2^ = 0.911 for E gene and r^2^ = 0.908 for RdRP gene), influenza A (r^2^ = 0.951) and influenza B (r^2^ = 0.978), while RSV showed moderate correlation with r^2^ = 0.772 (P<0.001 for all correlations). The slopes for all viruses were around 1, and their intercepts were also close to 1, ranging from -0.176 to 1.544.

Mean absolute bias and their limits of agreement measured by Bland-Altman analyses between C_t_ values obtained by AMPLIQUICK^®^ Respiratory Triplex and reference Allplex™ assays are depicted in Fig 1 for each target genes of SARS-CoV-2, influenza A/B and RSV. Finally, the mean absolute bias over the entire range of rtRT-PCR results for all viruses or target genes, was +0.665 C_t_ arbitrary unit (a.u.) (95% CI: 0.483–0.844) with limits of agreement from −5.311 to 3.981 C_t_ a.u.

## Discussion

In the present study, the novel multiplex rtRT-PCR assay AMPLIQUICK^®^ Respiratory Triplex for the simultaneous detection of SARS-CoV-2, influenza A, influenza B, and RSV showed high level of sensitivity, specificity, agreement, reliability and accuracy with reference comparator assays for the qualitative detection and differentiation of SARS-CoV-2, influenza A, influenza B, and RSV across a wide range of tested C_t_ values of the four viruses. Furthermore, high correlations of C_t_ values were observed for positive samples of the four viruses between the AMPLIQUICK^®^ Respiratory Triplex and reference comparator assays, with an overall high agreement between C_t_ values assessed by Bland-Altman analyses. This 96-well microplate rt RT-PCR associated with high-capacity automatic extractor provides a significant throughput in clinical biology. Overall, our observations indicate that the AMPLIQUICK® Respiratory Triplex assay is a reliable test for detecting SARS-CoV-2, influenza A, influenza B and RSV in respiratory samples. The analytical performance of this test appears to be of the same order as that of similar molecular methods currently used for the detection of these viruses in clinical samples. The ability to detect four respiratory viruses of interest in a single reaction makes it possible to rationalize and optimize the laboratory work and the management of patients suffering from respiratory manifestations in the context of influenza seasons.

There were only seven discordant samples for SARS-CoV-2, influenza A and influenza B, and these were resolved by third alternative assays. The discordant samples had high Ct values by the reference tests, which indicates that they probably had a low viral load or that the freeze-thaw steps could have damaged the nucleic acids in these samples, which resulted in negative impact on the detection of viral RNAs [38]. Furthermore, negative sample results cannot exclude inadequate sampling, or inadequate sample integrity of targets [39]. The introduction of the human RNase P gene as internal control in the AMPLIQUICK^®^ Respiratory Triplex allowed however confirming in part adequate sample specimens and appropriate testing conditions.

Even if patients infected with SARS-CoV-2, influenza A, influenza B or even RSV often present similar clinical pictures, their therapeutic management is quite different and specific for each respiratory viral infection. Molecular diagnostic tests quickly providing a reliable result constitute an important element in controlling these respiratory infections properly, and in adopting the best therapeutic choices. The SARS-CoV-2 pandemic has impacted algorithms for influenza-like-illness testing. During influenza seasons, molecular influenza diagnosis is recommended, especially if the patient is at risk of hospitalization or if the test result may impact patient management. [40]. From now on, the laboratory diagnosis of influenza during the flu winter season is become essential due to the circulation of SARS-CoV-2, overlapping clinical presentations of both influenza and COVID-19, as well as distinct infection control and public health ramifications of the two viruses in the setting of the global COVID-19 pandemic. Testing all patients presenting with influenza-like illness or COVID-19-like illness for relevant circulating respiratory viruses will be critical to the diagnosis and appropriate care of the patient, to identify co-infections, and to initiate appropriate public health surveillance and response to the on-going pandemic and collect accurate surveillance data. Otherwise, RSV detection is particularly valuable due to the seasonal overlap with influenza and similar symptoms in some patient populations including elderly and immunocompromised patients [41].

In addition, the periods of influenza-seasons aggravate the pressure on diagnostic laboratories, already acing unprecedented demand for molecular diagnostics due to the ongoing SARS-CoV-2 pandemic. Just like COVID-19, influenza is a major concern for infection control within healthcare facilities and symptoms are largely indistinguishable, particularly in the early phase of disease [42]. Consequently, SARS-CoV-2 and influenza viruses need to be concurrently tested for before contact precaution measures can be lifted for symptomatic patients. Finally, in light of the continuing worldwide shortage of supplies for nucleic acid extraction and PCR diagnostics, it appears desirable to be able to screen for all four viruses (SARS-CoV-2, influenza A, influenza B and RSV) within the same reaction.

Currently, multiple molecular diagnosis assays for SARS-CoV-2 RNA detection from rapid turnaround time to fully automated testing have been developed [43]. The association of the detection of SARS-CoV-2 with most other respiratory viruses quickly emerged from a syndromic diagnostic perspective. By this time, commercial providers have proposed multiplex molecular assays for numerous respiratory viruses, including SARS-CoV-2, such as single-use cassette with dedicated equipment [18, 19, 22] or multiplex rtRT-PCR assays usable on open system. However, the single-use cassette is high cost, with the requirement of dedicated instrument, and a low yield. Further, multiplex assays have been associated with higher reagent costs and lower sample processing capacity per day. Finally, the improvement of diagnostic testing was accomplished in practice by only diagnosing the four viruses of clinical interest. Thus, limited diagnosis strategy has been developed for simultaneous detecting SARS-CoV-2, influenza A, influenza B, and RSV, including in-house assays [44] as well as commercial assays, such as point-of-care rapid testing [20, 21] and high-throughput testing with open analyzers [23, 24]. Other options were the detection limited to SARS-CoV-2 and influenza viruses [45, 46]. The AMPLIQUICK^®^ Respiratory Triplex constitutes a solution adapted to the molecular diagnosis of the 4 viruses of interest, implantable on any open molecular biology platform, with manual or automatic extraction, at lower cost of reagents, with the possibility of series going up to 96 points in 3h30. As a result, implementing the multiplex AMPLIQUICK^®^ Respiratory Triplex is projected to have a positive impact during the winter seasons associated with the upfront simultaneous testing of SARS-CoV-2, influenza A and B, and RSV, in a context of rapid turn-around time. Using in routine the AMPLIQUICK^®^ Respiratory Triplex that targets all relevant respiratory viruses in the same well would allow for the detection of other viral infections in patients suspected of having COVID-19, that could expand the number of laboratories able to test for SARS-CoV-2 without ignoring coinfecting pathogens as alternative diagnoses for the decided treatment regimen.

Limitations of the study include the relatively small sample size for some viruses (influenza B and RSV). This was due to the small number of positive samples in the previous influenza-seasons in Paris and during the first and second waves of the COVID-19 epidemic in France. Second, this is a single-center study in which the AMPLIQUICK^®^ Respiratory Triplex assay needs to be further evaluated in other testing sites. Third, the study was retrospective and was conducted on frozen samples, which can lead to selection and sample quality bias.

## Conclusion

The overall performance of the AMPLIQUICK^®^ Respiratory Triplex was highly comparable to that of reference multiplex rtRT-PCR for the qualitative detection and differentiation of SARS CoV-2, influenza A, influenza B, and RSV in respiratory specimens. This novel assay may prove useful for streamlining diagnostics during the upcoming winter influenza-seasons.

## Supporting information

S1 Dataset Study’s minimal data set. (XLSX)

## References

[1] Zhou F, Yu T, Du R, Fan G, Liu Y, et al. Clinical course and risk factors for mortality of adult inpatients with COVID-19 in Wuhan, China: a retrospective cohort study. Lancet. 2020 Mar 28;395(10229):1054–1062. doi: 10.1016/S0140-6736(20)30566-3 32171076 PMC7270627

[2] Lansbury L, Lim B, Baskaran V, Lim WS. Co-infections in people with COVID-19: a systematic review and meta-analysis. J Infect. 2020;81(2):266–275. doi: 10.1016/j.jinf.2020.05.046 32473235 PMC7255350

[3] Petersen E, Koopmans M, Go U, Hamer DH, Petrosillo N, Castelli F, et al. Comparing SARS-CoV-2 with SARS-CoV and influenza pandemics. Lancet Infect Dis 2020; 20:e238–e244. doi: 10.1016/S1473-3099(20)30484-9 32628905 PMC7333991

[4] Solomon DA, Sherman AC, Kanjilal S. Influenza in the COVID-19 Era. JAMA. 2020 Oct 6;324(13):1342–1343. doi: 10.1001/jama.2020.14661 32797145

[5] Wu Z, McGoogan JM. Characteristics of and Important Lessons From the Coronavirus Disease 2019 (COVID-19) Outbreak in China: Summary of a Report of 72314 Cases From the Chinese Center for Disease Control and Prevention. JAMA 2020; doi: 10.1001/jama.2020.2648 32091533

[6] Azekawa S, Namkoong H, Mitamura K, Kawaoka Y, Saito F. Co-infection with SARS-CoV-2 and influenza A virus. IDCases. 2020 Apr 21;20:e00775. doi: 10.1016/j.idcr.2020.e00775 32368495 PMC7184249

[7] Cuadrado-Payán E, Montagud-Marrahi E, Torres-Elorza M, Bodro M, Blasco M, Poch E, et al. SARS-CoV-2 and influenza virus co-infection. Lancet 2020; 395:e84. doi: 10.1016/S0140-6736(20)31052-7 32423586 PMC7200126

[8] Hashemi SA, Safamanesh S, Ghafouri M, Taghavi MR, Mohajer Zadeh Heydari MS, Namdar Ahmadabad H, et al. Co-infection with COVID-19 and influenza A virus in two died patients with acute respiratory syndrome, Bojnurd, Iran. J Med Virol. 2020 Nov;92(11):2319–2321. doi: 10.1002/jmv.26014 32410338 PMC7272908

[9] Hirotsu Y, Maejima M, Shibusawa M, Amemiya K, Nagakubo Y, Hosaka K, et al. Analysis of Covid-19 and non-Covid-19 viruses, including influenza viruses, to determine the influence of intensive preventive measures in Japan. J Clin Virol. 2020;129:104543. doi: 10.1016/j.jcv.2020.104543 32663787 PMC7340051

[10] Huang BR, Lin YL, Wan CK, Wu JT, Hsu CY, Chiu MH, et al. Co-infection of influenza B virus and SARS-CoV-2: A case report from Taiwan. J Microbiol Immunol Infect. 2020 Jun 30;S1684-1182(20)30152-3. doi: 10.1016/j.jmii.2020.06.011 32646801 PMC7324913

[11] Kim D, Quinn J, Pinsky B, Shah NH, Brown I. Rates of Co-infection Between SARS-CoV-2 and Other Respiratory Pathogens. JAMA. 2020 May 26;323(20):2085–2086. doi: 10.1001/jama.2020.6266 32293646 PMC7160748

[12] Lai CC, Wang CY, Hsueh PR. Co-infections among patients with COVID-19: The need for combination therapy with non-anti-SARS-CoV-2 agents? J Microbiol Immunol Infect. 2020;53(4):505–512. doi: 10.1016/j.jmii.2020.05.013 32482366 PMC7245213

[13] Lu H, Stratton CW, Tang YW. Outbreak of pneumonia of unknown etiology in Wuhan, China: The mystery and the miracle. J Med Virol. 2020;92(4):401–402. doi: 10.1002/jmv.25678 31950516 PMC7166628

[14] Nowak MD, Sordillo EM, Gitman MR, Paniz Mondolfi AE. Coinfection in SARS-CoV-2 infected patients: Where are influenza virus and rhinovirus/enterovirus? J Med Virol. 2020 Oct;92(10):1699–1700. doi: 10.1002/jmv.25953 32352574 PMC7267652

[15] Wu X, Cai Y, Huang X, Yu X, Zhao L, Wang F, et al. Co-infection with SARS-CoV-2 and Influenza A Virus in Patient with Pneumonia, China. Emerg Infect Dis. 2020 Jun;26(6):1324–1326. doi: 10.3201/eid2606.200299 32160148 PMC7258479

[16] Yu F, Yan L, Wang N, Yang S, Wang L, Tang Y, et al. Quantitative Detection and Viral Load Analysis of SARS-CoV-2 in Infected Patients. Clin Infect Dis. 2020 Jul 28;71(15):793–798. doi: 10.1093/cid/ciaa345 32221523 PMC7184442

[17] Zheng X, Wang H, Su Z, Li W, Yang D, Deng F, et al. Co-infection of SARS-CoV-2 and Influenza virus in Early Stage of the COVID-19 Epidemic in Wuhan, China. J Infect. 2020 Aug;81(2):e128–e129. doi: 10.1016/j.jinf.2020.05.041 32474045 PMC7255712

[18] Brendish NJ, Poole S, Naidu VV, Mansbridge CT, Norton NJ, Wheeler H, et al. Clinical impact of molecular point-of-care testing for suspected COVID-19 in hospital (COV-19POC): a prospective, interventional, non-randomised, controlled study. Lancet Respir Med. 2020 Dec;8(12):1192–1200. doi: 10.1016/S2213-2600(20)30454-9 33038974 PMC7544498

[19] Creager HM, Cabrera B, Schnaubelt A, Cox JL, Cushman-Vokoun AM, Shakir SM, et al. Clinical evaluation of the BioFire® Respiratory Panel 2.1 and detection of SARS-CoV-2. J Clin Virol. 2020 Aug;129:104538. doi: 10.1016/j.jcv.2020.104538 32650276 PMC7336953

[20] Leung EC, Chow VC, Lee MK, Tang KP, Li DK, Lai RW. Evaluation of the Xpert Xpress SARS-CoV-2/Flu/RSV assay for simultaneous detection of SARS-CoV-2, Influenza A/B and Respiratory Syncytial Viruses in nasopharyngeal specimens. J Clin Microbiol. 2021 Jan 12:JCM.02965-20. 10.1128/JCM.02965-20 PMC8092745 33436456

[21] Mostafa HH, Carroll KC, Hicken R, Berry GJ, Manji R, Smith E, et al. Multi-center Evaluation of the Cepheid Xpert® Xpress SARS-CoV-2/Flu/RSV Test. J Clin Microbiol. 2020 Dec 9:JCM.02955-20.

[22] Visseaux B, Le Hingrat Q, Collin G, Bouzid D, Lebourgeois S, Le Pluart D, et al. Evaluation of the QIAstat-Dx Respiratory SARS-CoV-2 Panel, the First Rapid Multiplex PCR Commercial Assay for SARS-CoV-2 Detection. J Clin Microbiol. 2020 Jul 23;58(8):e00630–20. doi: 10.1128/JCM.00630-20 32341142 PMC7383528

[23] Chung HY, Jian MJ, Chang CK, Lin JC, Yeh KM, Chen CW, et al. Novel dual multiplex real-time RT-PCR assays for the rapid detection of SARS-CoV-2, influenza A/B, and respiratory syncytial virus using the BD MAX open system. Emerg Microbes Infect. 2021 Dec;10(1):161–166. doi: 10.1080/22221751.2021.1873073 33410371 PMC7832498

[24] Nörz D, Hoffmann A, Aepfelbacher M, Pfefferle S, Lütgehetmann M. Clinical evaluation of a fully automated, laboratory-developed multiplex RT-PCR assay integrating dual-target SARS-CoV-2 and influenza A/B detection on a high-throughput platform. J Med Microbiol. 2021 Jan 6. doi: 10.1099/jmm.0.001295 33404401 PMC8131019

[25] Gimferrer L, Andrés C, Rando A, Piñana M, Codina MG, Martin MDC, et al. Evaluation of Seegene Allplex Respiratory Panel 1 kit for the detection of influenza virus and human respiratory syncytial virus. J Clin Virol. 2018 Aug;105:31–34. doi: 10.1016/j.jcv.2018.05.006 29883908 PMC7106510

[26] Farfour E, Lesprit P, Visseaux B, Pascreau T, Jolly E, Houhou N, et al. SARS-CoV-2 Foch Hospital study group. The Allplex 2019-nCoV (Seegene) assay: which performances are for SARS-CoV-2 infection diagnosis? Eur J Clin Microbiol Infect Dis. 2020 Oct;39(10):1997–2000. doi: 10.1007/s10096-020-03930-8 32462501 PMC8824685

[27] Newcombe RG. Two-sided confidence intervals for the single proportion: comparison of 362 seven methods. Statistics in Medicine. 1998;17:857–872. doi: 10.1002/(sici)1097-0258(19980430)17:8&lt;857::aid-sim777&gt;3.0.co;2-e 9595616

[28] Cohen J. A coefficient of agreement for nominal scales. Educ. Psychol. Meas. 1960;20:37–46.

[29] Landlis JR, Koch GG. The measurement of observer agreement for categorical data. Biometrics. 1977;33(1):159–74. 843571

[30] Youden WJ. Index for rating diagnostic tests. Cancer.1950;3(1):32–35. doi: 10.1002/1097-0142(1950)3:1&lt;32::aid-cncr2820030106&gt;3.0.co;2-3 15405679

[31] Bénézit F, Loubet P, Galtier F, Pronier C, Lenzi N, Lesieur Z, et al. Non-influenza respiratory viruses in adult patients admitted with influenza-like illness: a 3-year prospective multicenter study. Infection. 2020 Aug;48(4):489–495. doi: 10.1007/s15010-019-01388-1 32056143 PMC7095392

[32] Passing H, Bablok. A new biometrical procedure for testing the equality of measurements from two different analytical methods. Application of linear regression procedures for method comparison studies in clinical chemistry, Part I. J Clin Chem Clin Biochem. 1983, 21 (11): 709–720. doi: 10.1515/cclm.1983.21.11.709 6655447

[33] Bland JM, Altman DG. Statistical methods for assessing agreement between two methods of clinical measurement. Lancet. 1986, 1 (8476): 307–310. 2868172

[34] Bland JM, Altman DG. Measuring agreement in method comparison studies. Stat Methods Med Res. 1999, 8 (2): 135–160. doi: 10.1177/096228029900800204 10501650

[35] Journal Officiel de la République Française. Ordonnance n° 2010–49 du 13 janvier 2010 relative à la biologie médicale. Available from: https://www.legifrance.gouv.fr/jorf/id/JORFTEXT000021683301/ (last access 31 January 2021).

[36] Centers for Disease Control and Prevention. Common Investigation Protocol for Investigating Suspected SARS-CoV-2 Reinfection. 2020. Available at: https://www.cdc.gov/coronavirus/2019-ncov/php/reinfection.html (last access 21 January 2021).

[37] Jefferson T, Spencer EA, Brassey J, Heneghan C. Viral cultures for COVID-19 infectious potential assessment—a systematic review. Clin Infect Dis. 2020 Dec 3:ciaa1764. 10.1093/cid/ciaa1764 PMC7799320 33270107

[38] Yu K, Xing J, Zhang J, Zhao R, Zhang Y, Zhao L. Effect of multiple cycles of freeze thawing on the RNA quality of lung cancer tissues. Cell Tissue Bank 2017;18:433–440. doi: 10.1007/s10561-016-9600-7 28573389

[39] Brukner I, Eintracht S, Papadakis AI, Faucher D, Lamontagne B, Spatz A, et al. Maximizing confidence in a negative result: Quantitative sample adequacy control. J Infect Public Health 2020; 13:991–993. doi: 10.1016/j.jiph.2020.01.307 32037201

[40] Centers for Disease Control and Prevention. COVID-19: CDC guidance for expanded screening testing to reduce silent spread of SARS-CoV-2. Atlanta, GA: US Department of Health and Human Services; 2020. Available at: https://www.cdc.gov/coronavirus/2019-ncov/php/open-america/expanded-screening-testing.html (last access 21 January 2021).

[41] Uyeki TM, Bernstein HH, Bradley JS, Englund JA, File TM, Fry AM, et al. Clinical Practice Guidelines by the Infectious Diseases Society of America: 2018 Update on Diagnosis, Treatment, Chemoprophylaxis, and Institutional Outbreak Management of Seasonal Influenzaa.Clin Infect Dis. 2019 Mar 5;68(6):e1–e47. doi: 10.1093/cid/ciy866 30566567 PMC6653685

[42] Wu D, Lu J, Ma X, Liu Q, Wang D, Gu Y, et al. Coinfection of Influenza Virus and Severe Acute Respiratory Syndrome Coronavirus 2 (SARS-COV-2). Pediatr Infect Dis J. 2020 Jun;39(6):e79. doi: 10.1097/INF.0000000000002688 32287051 PMC7258765

[43] Tang YW, Schmitz JE, Persing DH, Stratton CW. Laboratory Diagnosis of COVID-19: Current Issues and Challenges. J Clin Microbiol 2020; 58:e00512–20. doi: 10.1128/JCM.00512-20 32245835 PMC7269383

[44] Centers for Disease Control and Prevention.2020. Research Use Only CDC Influenza SARS-CoV-2 (Flu SC2) Multiplex Assay Real-Time RT-PCR Primers and Probes. Available at: https://www.cdc.gov/coronavirus/2019-ncov/lab/multiplex-primer-probes.html (last access 07 February 2021).

[45] Mancini F, Barbanti F, Scaturro M, Fontana S, Di Martino A, Marsili G, et al. Multiplex rt-Real Time PCR assays for diagnostic testing of SARS-CoV-2 and seasonal influenza viruses. A challenge of the phase 3 pandemic setting. J Infect Dis. 2020 Oct 20:jiaa658. 10.1093/infdis/jiaa658 PMC7665649 33080031

[46] Ni M, Xu H, Luo J, Liu W, Zhou D. Simultaneous detection and differentiation of SARS-CoV-2, influenza A virus and influenza B virus by one-step quadruplex real-time RT-PCR in patients with clinical manifestations. Int J Infect Dis. 2020 Dec 14;103:517–524. doi: 10.1016/j.ijid.2020.12.027 33326873 PMC7836965

